# An Authentic, Practice-Based Assessment as a Catalyst for Continuous Professional Development

**DOI:** 10.3390/pharmacy8010015

**Published:** 2020-02-04

**Authors:** Sandra Winkelbauer

**Affiliations:** Ontario College of Pharmacists, Registrant Competence, Toronto, ON M5R 2R4, Canada; swinkelbauer@ocpinfo.com

**Keywords:** practice-based assessment, continuing professional development, quality assurance, regulatory body, continuing education, competency, pharmacists

## Abstract

Over the last ten years, pharmacy practice has changed significantly in Canada. It is more important than ever to ensure that the profession engages in continuing professional development in order to keep up with changing practice and changing public demand and scrutiny. The question is, how do we ensure that the required continual professional development occurs and is applied to practice? One Canadian regulator, the Ontario College of Pharmacists, has attempted to address this question by assessing the success of a number of quality assurance options in terms of addressing the competence of pharmacists, and by extension their ability to learn and apply their learning in an ongoing manner. This case study presents three policy options; an analysis of those options; and finally, an evaluation of the best option for this regulator. The policy alternatives considered include a continuing education/professional development requirement, standardized simulated assessment (i.e., observed structured clinical examination) and authentic practice-based assessment. For the Ontario College of Pharmacists, an authentic practice-based assessment approach seems effective at stimulating quality improvements in pharmacists’ practice, likely because the assessment acts as a catalyst for pharmacists to engage in continuing professional development in order to maintain competence.

## 1. Introduction

Regulated health professionals are entrusted with the responsibility to care for patients [1]. Regulatory bodies, which are agencies responsible for regulating health professionals, are mandated to ensure patient safety. Regulatory bodies achieve this mandate through three main activities or programs: registration/licensing, quality assurance, and complaints/discipline [2].

Registration requirements, which generally include formal education, entry-to-practice examinations and practical experience, aim to ensure that an applicant has the required competencies to practice patient care prior to licensing [1,2]. Quality assurance activities are intended to ensure that professionals continue to be competent throughout their careers. This is especially critical for health professionals, because medical knowledge changes so rapidly, with some reports indicating a half-life of about five years [1]. The complaints/disciplinary processes of regulatory bodies address any concerns that patients or the public have with respect to individual health professionals [2].

Much focus is put on evaluating whether an applicant has the required knowledge, skills and behaviour to be licensed or registered [1]. That is critical; however, these registered health professionals will likely be practicing the profession for decades. The emphasis on initial competence and lack of emphasis on life-long competence has recently been questioned more and more [1,3].

In addition, pharmacy practice is changing. With more complex care requirements, healthcare has had to evolve. Part of this evolution is a changing role for the pharmacist. Increasingly, pharmacists are being recognized as medication management experts and have new responsibilities with respect to the care of the patient. Over the last ten years, many provinces in Canada have added prescriptive authority (both for initial prescriptions of various types and adaptation/management of current prescriptions), injection authority and authority to order and interpret laboratory tests [4]. With these increasing responsibilities, there is also increasing public demand to ensure that practices are appropriate and safe.

In this environment, continuing professional development is crucial. If pharmacists are not continually upgrading their knowledge and skills and applying these to practice, they will not meet standards for present-day care. The question is, how do we ensure that the required continuing professional development occurs and is applied in practice?

## 2. Problem Definition

In Ontario, all regulatory bodies for health professions must have a quality assurance program [5]. As described by Austin et al., the mandates of these quality assurance programs are, “To ensure professionals are engaged in a process of life-long learning so that, at all stages of their careers, they continue to possess the knowledge, skills and judgement necessary to competently practice their profession; thus, ultimately ensuring protection of the public [1].” The problem is that there is great variation in quality assurance programs. For many programs, there is a lack of evidence for the efficacy of the program. There is no consensus on the ideal program, and there are limitations such as cost which may restrict the type of program provided by the regulators [1,3,6,7]. Because of these shortcomings, quality assurance programs are not effectively giving the public confidence in the competence of their health care professionals [1,3].

Thus, the question that both the public and regulators would like to have answered is: “What is the most effective type of quality assurance program to ensure that healthcare professionals engage in continuing professional development and apply learning to practice?” Further, regulators also want to know what the most cost-effective approach is.

## 3. Case Study—Ontario College of Pharmacists

One Canadian regulator, the Ontario College of Pharmacists (Appendix A), has attempted to address the above questions by assessing the success of a number of quality assurance options which address the competence of pharmacists, and by extension their ability to learn and apply their learning in an ongoing manner. Specifically, the Ontario College of Pharmacists assessed the efficiency, addressing both effectiveness and cost, of three quality assurance options. The following case study presents the policy options considered; an analysis of those options; and finally, an evaluation of the best option for this regulator.

## 4. Policy Options

This analysis examines three policy alternatives to address the need for a quality assurance program for pharmacists: continuing education/professional development requirement, standardized simulated assessment (i.e., observed structured clinical examination—OSCE) and authentic practice-based assessment.

### 4.1. Continuing Education/Professional Development Requirement

Most regulatory bodies require ongoing learning for maintenance of competence; however, often this is just one component of a multi-pronged approach [1]. In the United States, continuing education/professional development requirement programs (plus or minus a self-assessment component), are in most jurisdictions, the primary or only quality assurance activity for pharmacy professionals [3,8]. For this reason, this option was chosen as a comparator. Essentially practitioners are required to engage in learning for a specified period of time or a specified number of contact hours [9]. Generally, the learning has to be documented in a learning portfolio or some similar tool.

### 4.2. Standardized Simulated Assessment

A standardized simulated assessment, often referred to as an observed structured clinical examination (OSCE), is an assessment where a practitioner is exposed to a standardized case and is evaluated on their ability to provide patient care [1,10]. The Ontario College of Pharmacists used an OSCE as part of its quality assurance program for almost twenty years [10].

### 4.3. Authentic, Practice-Based Assessment

In this assessment, trained peers evaluate a practitioner under naturalistic (e.g., in their place of practice) conditions through observation and chart-stimulated recall [1,2,11]. This assessment approach has been used by the Ontario College of Pharmacists for the past three years (Appendix B).

## 5. Analysis

### 5.1. Continuing Education/Professional Development Requirement

Intuitively, the requirement for continuing education/professional development makes sense, given the rapidly changing body of knowledge in healthcare. As a result, as described above, most regulatory bodies have a requirement to engage in some form of life-long learning [1]. Although this requirement is part of most quality assurance programs, many regulatory bodies have this requirement as their only or their primary quality assurance activity [3,9].

A primary advantage of a quality assurance program that is centred on a continuing education/professional development requirement is accessibility or reach. Because this type of quality assurance activity is relatively easy to administer and low in costs, many or all practitioners can participate. Austin et al., in their review of 91 regulatory bodies, found that 68 of them required minimum mandatory education [1]. The median requirement was approximately 25 h or units per year. Generally, practitioners are offered choices of activities and learning methods [1]. From the perspective of the regulatory bodies, this option requires minimal resources. Some regulatory bodies require an annual declaration that the requirement was met; others require documentation of the learning programs completed in a learning portfolio or something similar; and others still have an audit process [3,8,9]. Regardless of the approach taken, all practitioners can participate in this quality assurance activity every year without an undue constraint on resources for the regulatory body. (If an audit process is implemented, there is an impact on resources that will be proportional to the percentage of practitioners audited.)

To provide an estimate of costs, experience from one regulatory body, the Ontario College of Pharmacists, is highlighted here. The Ontario College of Pharmacists has required continuing education/professional development for over twenty years. Pharmacists are required to engage in learning and document the learning in a learning portfolio; however, a specific number of hours or units is not required; there are no specifications regarding the type of learning activity and an audit process is not used [2]. The costs borne by the regulatory body relate to the provision of an online Continuing Professional Development Portal where pharmacists can access a self-assessment tool, identify their learning needs and document the learning that has been completed. The annual cost related to maintenance of this online tool is approximately CAN$24,000. This allows access for 6000 users. An additional charge is levied if there are more than 6000 users; however, since pharmacists are also able to use other means of documenting their learning, the additional charge has not been billed [8].

With respect to fairness or equitability, this type of quality assurance activity scores high marks. The requirements are the same for everyone, and because the choice of education can be adapted to the individual user’s unique practice, the activity cannot be criticized as being too general or too specific [9].

The primary disadvantage of this type of program is a lack of proven effectiveness [12,13]. As Austin et al. note, “There is virtually no evidence available to support this practice or to establish any correlation to positive, practice-related outcomes.” In addition, the authors indicate that there is some evidence that compulsory education has minimal or no effect on professional behavioural changes, and one study found that requiring continuing education does not improve performance in incompetent individuals [1]. Even for those programs that have evolved to a continuing professional development (CPD) model, incorporating a learning cycle and/or self- assessment, no evidence was found supporting efficacy [1].

### 5.2. Standardized Simulated Assessment

Although observed structured clinical examinations (OSCEs) can vary, there are generally a number of standardized cases, with trained standardized actors, and trained peer assessors evaluating the interactions [10]. The assessors use a standardized assessment tool with specified criteria. A psychometric process is used to determine an appropriate cut score to differentiate those that have the required competencies from those that require remediation [10].

One advantage of an OSCE is that there is evidence to support the use of this type of quality assurance activity in providing assurance of ongoing competence of practitioners [1,14]. As described by Austin et al., a simulation-based assessment has the ability to evaluate a practitioner’s performance, and as such it is a higher level of assessment than other types of quality assurance activities [9]. In addition, because of the psychometric foundation and standardized processes, OSCEs have proven to be a valid and reliable assessment method [9]. Having said this, there are also some limitations with respect to effectiveness. Specifically, fidelity with real world experience is a commonly cited limitation [9]. Although great effort goes into trying to create simulations that are reflective of reality, in the end, they are still simulated examination settings. In the context of pharmacists, one is removing many of the external factors that might influence performance in their real-life settings—both in a positive and negative manner. So, although performance is assessed with an OSCE, the assessment is unable to identify whether a practitioner would perform in the same way in their own practice setting [9].

In order to ensure defensibility of this quality assurance activity, regulators expend significant resources to ensure the assessment is valid and reliable. For this reason, it is fair for practitioners in that they all receive an equal assessment. Having said this, based on feedback from pharmacists that participated in the Ontario College of Pharmacists’ OSCE, some pharmacists perceived an element of inequity because the assessment is based in general practice and those with a specialty practice may feel less familiar and less comfortable. In general though, pharmacists indicated that they found the OSCE useful and acceptable [8].

As described above, the resources required by the regulator to provide this type of quality assurance activity are significant. As a result, accessibility is limited, and is a major limitation for this type of activity. To provide an estimate of costs and numbers of pharmacists that might be assessed, the experience of the Ontario College of Pharmacists with the provision of an OSCE from 1997 to 2016 is highlighted here.

In order to provide the assessment, the following costs were incurred:

- Development of cases with peer pharmacists;
- Standard setting for cases with peer pharmacists;
- Training for standardized patients and assessors;
- Standardized patients and assessors for duration of assessment;
- Psychometric services;
- Administrative costs.

As an example, in 2011, approximately CAN$450,000 was spent on the quality assurance program, with the majority being spent on the OSCE. (Note that this excludes staffing for the program). In 2011, approximately 280 pharmacists were assessed, with about 240 being initial assessments and the remainder being reassessments. Thus, the cost per pharmacist assessed (excluding administrative staff) was approximately CAN$1600. (Approximately CAN$1800 in today’s dollars) [8,15].

In 2011, there were approximately 11,600 practicing pharmacists in Ontario [16]. Therefore, approximately two percent of practicing pharmacists in Ontario were assessed. So, even though the OSCE is an efficacious, valid and reliable assessment, the limited reach, due to cost restraints, is an issue and impacts the overall effectiveness of this type of quality assurance activity in ensuring competence of all practicing pharmacists.

### 5.3. Authentic, Practice-Based Assessment

An authentic, practice-based assessment, for the purposes of this review, is defined as a combination of direct observation and chart-stimulated recall which takes place in the practitioner’s place of practice. Direct observation, as the name implies, refers to observing a practitioner providing patient care in their own practice setting [9]. Chart stimulated recall involves questioning the practitioner about thought processes in the provision of care to their patients [9]. This assessment method often employs behavioural-based interviewing—which is based on the concept that past behaviour predicts future behaviour. Using examples from the practitioner’s practice, the healthcare professional is asked questions regarding their processes and rationale for decisions made [9,11].

The prime advantage of an authentic, practice-based assessment is its effectiveness in a changing practice, and therefore, improving care provided to patients. To address the effectiveness of this process, the effectiveness of both direct observation and chart stimulated recall have to be reviewed.

Direct observation is considered as a valuable tool with good content validity by psychometricians, because practitioners are being assessed in a “real” environment with “real” patients. One variation of direct observation is concealed observation, where the practitioner does not know that they are being observed and assessed. There is some evidence from a “mystery shopper” program in Australia that indicates that this type of assessment has consequential validity and contributes to practice improvement [9,17]. (However, as described above, acceptability is an issue with this type of direct observation.)

Many components of competency can be evaluated through a chart review-based assessment. Beyond these components, a chart-stimulated recall type of assessment can provide insight into behaviour, judgement, decision-making and application of knowledge [9].

With the process used by the Ontario College of Pharmacists, feedback and coaching are provided during and after the assessment in all areas where there is a potential for improvement [11]. Because some evidence suggests that immediate follow-up is important in influencing professional improvement, this timely feedback is important [9]. In addition, this process is especially effective because it relates to actual issues occurring in the practitioner’s place of practice rather than simulated scenarios in a sterilized environment.

Some sources indicate that the Hawthorne effect, which is defined as “the stimulation to output or accomplishment that results from the mere fact of being under observation,” might be a limitation for authentic practice-based assessment because professionals may be on their best behaviour while being observed [9,18]. However, the opposite might also be true; that is, the Hawthorne effect may actually contribute to the effectiveness of this model. In other words, the fact that one will be assessed in their place of practice may cause practice change in preparation.

Another possible limitation is the extent of standardization. Unlike the standardized simulated assessment, an authentic, practice-based assessment relies on real cases rather than standardized cases. Performance on these real cases may be affected by external factors, and as a result, the generalizability of the results may be limited. The best way to counter this limitation is to review a sample of charts and cases and to provide adequate training for assessors [9].

Although the authentic, practice-based assessment does not have standardized cases, other elements of the assessment are often standardized [11]. In the Ontario College of Pharmacists’ model, all practitioners undergo a similar review process, and therefore, there is procedural fairness. In addition, standardized assessment tools, with an emphasis on the training of peer assessors and psychometric methods, provide further standardization, and improve the reliability and validity of assessments and the defensibility of the process [9,11].

The Ontario College of Pharmacists has used an authentic, practice-based assessment for the last three years. Preliminary results, available for the last three years, indicate effectiveness with a gradual improvement in practice (see Table 1) [8,19]. Note that different cohorts or pharmacists were assessed each year.

Similar to the standardized simulated assessment, the resources required by the regulator to provide this type of quality assurance activity are significant. The Ontario College of Pharmacists’ program is a modified version of an authentic practice-based assessment. Rather than using full time practitioners as peer assessors, the College chose to use pharmacists practicing part-time (usually 1–2 days per week) so that the remainder of the work week could be focused on assessments. As a result, the College has a small pool of trained peer assessors on staff. The majority of costs incurred from this quality assurance activity relate to the pool of assessors:

- Salaries for peer assessors;
- Benefits for peer assessors;
- Travel costs, including meals and accommodation.

Using 2017 as a representative example, approximately CAN$1,278,000 was spent on salaries and benefits for peer assessors and about CAN$180,000 was spent on travel costs for these peer assessors [8]. In 2017, 2673 assessments were conducted [19]. Thus, the cost per pharmacist assessed (excluding administrative staff) was about CAN$545. Although this is costly, it is much less expensive than the standardized simulated assessment approach. In addition, these assessors are currently also tasked with assessing the pharmacies. If this activity were to be transferred to other staff, the peer assessors might be able to significantly increase the number of pharmacist assessments completed in a given time period. Ultimately, this would reduce the cost per assessment [8].

In 2017, there were 15,192 practicing pharmacists in Ontario [19]. Therefore, approximately 18 percent of practicing pharmacists in Ontario were assessed. So, even though there are some limitations with an authentic practice-based assessment, the benefits with respect to effectiveness and reach (or access), make this type of quality assurance activity beneficial in ensuring competence of all practicing pharmacists.

With respect to acceptability of an authentic practice-based assessment, satisfaction surveys indicated that pharmacists found this type of quality assurance activity acceptable and useful in improving practice [8]. The following quotes from pharmacists that participated in an authentic practice-based assessment illustrate their favourable reactions [20]:

- “It was a chance for self-reflection and positive reinforcement of everything we are doing well. It was a two-way conversation with another pharmacist/educator who had great ideas and insight into the reasons why we do things and how to improve on them.”
- “Here’s what we’re doing well, and here’s where we can do even better.”
- “The entire experience was very positive and will translate to even better patient care.”
- “I like the spirit of this practice assessment. We’re being asked to reflect on and talk through our processes. For instance, I had to show a medication review, and the advisor asked me about my rationale for the steps I took.”
- “Pharmacists become accustomed to doing the same things every day. You get in the habit of just doing something a certain way. When you start understanding the ‘why’ of what you’re doing, it takes hold and you can be more insightful. When you boil it all down, the reason we’re here is to improve patient outcomes.”

## 6. Evaluation

As indicated earlier, with respect to quality assurance, ultimately, the goal of the public and regulators is to ensure that healthcare professionals engage in continuing professional development and apply learning to practice. Each of the quality assurance activities described have pros and cons with respect to this outcome and each might be the most appropriate choice in certain circumstances.

For the Ontario College of Pharmacists, an authentic practice-based assessment as a quality assurance activity seems to be the ideal choice. The authentic practice-based assessment has proven to have considerable reach (reaching approximately 18% of practicing pharmacists per year in the Ontario College of Pharmacists’ model), and at the same time, has demonstrated positive outcomes by stimulating quality improvement. In addition, although this option does not provide the same standardization as the standardized simulated assessment, it does incorporate enough standardization to make it a defensible assessment. Finally, with respect to acceptability among pharmacists, anecdotal reports indicate that pharmacists are engaged by this type interaction and find it acceptable.

For other regulators, different priorities might lead to the selection of a different quality assurance activity as the ideal choice. For example, if engaging every practitioner every year is the priority, the continuing education/professional development option would likely be chosen. Or, if ensuring rigorous standardization is the priority, the standardized simulated assessment would be a more ideal option.

## 7. Discussion

Healthcare information is constantly changing. In fact, some references indicate that these days, medical knowledge has a very short half-life, as little as five years [1]. Because of this, continuing professional development is especially important in the health professions.

The consequences of not engaging in ongoing learning can be significant. In more extreme cases, these practitioners will practice the way they did when they graduated. Although standards evolve and medical information evolves, their practice does not evolve, and they fall further behind every year. Although this is an extreme case, a milder version might be seen with a greater percentage of practitioners as they move along their career path and other responsibilities encroach on their responsibility for continuous professional development.

The question is, “For that practitioner that is not engaged or minimally engaged in continuous professional development, what can be done to encourage ongoing learning and practice change?” A quality assurance activity such as an authentic practice-based assessment will act as a catalyst for learning, because most practitioners will want to be prepared prior to undergoing an assessment and because practitioners are more likely to learn when engaged. In the case study presented, the authentic practice-based assessment approach was preferred because it reached a greater number of practitioners (thus having a more significant impact on the profession) and because it took place in the practitioner’s place of practice, making practice changes more likely.

As illustrated by the case study, the authentic practice-based assessment was effective in stimulating quality improvement for pharmacists in Ontario. Over three years, the percentage of pharmacists meeting standards in more than half of the performance indicators increased and the percentage of pharmacists meeting standards in all performance indicators increased. In addition, based on feedback from pharmacists that were assessed, in general, pharmacists appreciated this form of assessment. For those who met standards, the authentic practice-based assessment validated their work. For those that met some standards but had opportunities for improvement in others, the authentic practice-based assessment resulted in recommendations for continuing education—which the pharmacists were likely to engage in because it was relevant to their particular practice and it addressed an issue that would be assessed again at some point in the future. In other words, this process provided an incentive or catalyst for the pharmacist to engage in continuing professional development. For those that did not meet standards, specified remediation was required. As a result, this process encouraged ongoing learning and practice changes for pharmacists all along the continuum of practice (exemplary practice to remedial practice), and therefore, was effective in stimulating quality improvement.

The impact of this type of assessment process for educators is significant. The most important aspect is that more practitioners will likely engage in important continuing professional development. Thus, demand for practice-relevant, quality education is expected to increase.

## 8. Limitations

Although there is an impact in terms of quality improvement with an authentic practice-based assessment, there are a number of limitations in demonstrating effectiveness. Ideally, effectiveness would be measured using patient outcomes; however, at this time, this is not feasible in the complex world of healthcare, wherein pharmacy is one part. In the future, with evolving data analytics, it is hoped that this question will be addressed and that predictive validity of this type will be confirmed.

Effectiveness could also be inferred from a correlation of performance on the assessment to data such as complaints or disciplinary instances. At this point, there is insufficient data to perform this type of predictive analysis; however, this might also be possible in the future. Assessment of concurrent validity, comparing performance on the assessment to other forms of recognition (such as awards for practice), is also beyond current scope.

In summary, it is acknowledged that there are limitations in determining both predictive validity and concurrent validity. This case study provides a good starting point for future research in this area.

## 9. Conclusions

As described above, over the last ten years, pharmacy practice has changed significantly. It is more important than ever to ensure that the profession engages in continuing professional development in order to keep up with changing practice and changing public demand and scrutiny.

Based on this case study, an authentic practice-based assessment approach seems effective in stimulating quality improvement in pharmacists’ practice because the assessment acts as a catalyst for pharmacists to engage in continuing professional development in order to maintain competence. Further research is required to confirm these initial findings and to address the limitations identified.

## Figures and Tables

**Table 1:** Pharmacists’ performances in an authentic, practice-based assessment.

	2017	2018	2019 *
Percentage of pharmacists meeting standards in more than half of the performance indicators	74.1%	77.7%	81.0%
Percentage of pharmacists meeting standards in all performance indicators	8.4%	8.9%	12.5%
Percentage of pharmacists not meeting standards in one or more performance indicators	3.4%	2.2%	1.9%

* 2019 January to September.

## Appendix A

The Ontario College of Pharmacists (OCP), incorporated in 1871, is the registering and regulating body for the profession of pharmacy in Ontario. The College’s mandate is to serve and protect the public and hold Ontario’s pharmacists and pharmacy technicians accountable to the established legislation, standards of practice, code of ethics and policies and guidelines relevant to pharmacy practice. The College also oversees the province’s community and hospital pharmacies and assesses them against prescribed standards of operation. Over 16,500 pharmacists, and over 4500 pharmacy technicians, are registered in Ontario. Approximately 4500 pharmacies are accredited. For more information, visit www.ocpinfo.com.

## Appendix B

Adapted with permission from the Ontario College of Pharmacists website (January 16, 2020): Practice Assessment Summary [21].

A practice assessment is an evaluation of an individual pharmacist’s or pharmacy technician’s performance. The practice assessment occurs in the place of practice with an OCP practice advisor. Practice assessments are separate and distinct from a pharmacy assessment.

Practice assessment of pharmacists support their role as medication experts and clinical decision-makers, and are consistent in approach to the assessments of other primary healthcare practitioners.

Practice assessments are a critical component of quality assurance. The transition to assessments at the individual’s place of practice reflects evolving public and patient expectations that the College regularly engages with pharmacists and pharmacy technicians to ensure that safe and appropriate patient care is being provided.

Ultimately, quality assurance activities are designed to help pharmacy professionals identify areas of improvement in their practice so that they can develop, maintain and enhance their skills and knowledge to support better patient health outcomes.

During a practice assessment, practice advisors use the practice assessment criteria to evaluate a registrant’s practice. For pharmacists, practice advisors focus on four key areas: patient assessment, decision making, documentation and communication/education.

Throughout and following the assessment, the practice advisor provides feedback outlining areas of practice where the pharmacy professional is doing well and meeting standards, and areas where there is an opportunity for improvement. They offer support through coaching and conversation—pointing out opportunities to enhance practice, probing the thinking behind certain actions and decisions and indicating where to access helpful resources.

If a pharmacy professional does not meet the standards indicated on their first assessment, they are given the opportunity to spend time with a quality assurance (QA) coach. This coach is not a College staff member, but rather a peer who can provide support specifically in areas where there is room for improvement. This half-day interactive session is designed to enhance the professional’s practice and the care that is provided to patients. Following the session with the QA coach, the pharmacy professional will be reassessed by a separate practice advisor.

If there are still significant areas of practice that require improvement following this second assessment, a QA assessment will take place and the results will be sent to the QA Committee for consideration. The QA Committee may provide recommendations to help the professional meet standards by identifying appropriate remediation, always recognizing that patient safety is the first priority.

With an emphasis on education, the goal of the practice assessments is to increase adherence to practice standards; help pharmacy professionals practice to their full capabilities; and ultimately, support optimal health outcomes. The results of a practice assessment are confidential and are not shared with employers, owners, colleagues or any College committee, other than the QA Committee.
